# Evaluation of a Bayesian penalized likelihood reconstruction algorithm for low-count clinical ^18^F-FDG PET/CT

**DOI:** 10.1186/s40658-019-0262-y

**Published:** 2019-12-30

**Authors:** Joost te Riet, Sjoerd Rijnsdorp, Mark J. Roef, Albert J. Arends

**Affiliations:** 1 Department of Medical Physics, Catharina Hospital Eindhoven, Eindhoven, The Netherlands; 2 Department of Radiology and Nuclear Medicine, Radboud University Medical Center, Nijmegen, The Netherlands; 3 Department of Medical Physics, Radboud University Medical Center, Nijmegen, The Netherlands; 4 Department of Nuclear Medicine, Catharina Hospital Eindhoven, Eindhoven, The Netherlands

**Keywords:** Bayesian penalized likelihood, NEMA image quality phantom, Micro Hollow Sphere phantom, image quality, image reconstruction, optimization, positron emission tomography, Q.Clear

## Abstract

**Background:**

Recently, a Bayesian penalized likelihood (BPL) reconstruction algorithm was introduced for a commercial PET/CT with the potential to improve image quality. We compared the performance of this BPL algorithm with conventional reconstruction algorithms under realistic clinical conditions such as daily practiced at many European sites, *i.e.* low ^18^F-FDG dose and short acquisition times.

**Results:**

To study the performance of the BPL algorithm, regular clinical ^18^F-FDG whole body PET scans were made. In addition, two types of phantoms were scanned with 4-37 mm sized spheres filled with ^18^F-FDG at sphere-to-background ratios of 10-to-1, 4-to-1, and 2-to-1. Images were reconstructed using standard ordered-subset expectation maximization (OSEM), OSEM with point spread function (PSF), and the BPL algorithm using β-values of 450, 550 and 700. To quantify the image quality, the lesion detectability, activity recovery, and the coefficient of variation (COV) within a single bed position (BP) were determined. We found that when applying the BPL algorithm both smaller lesions in clinical studies as well as spheres in phantom studies can be detected more easily due to a higher SUV recovery, especially for higher contrast ratios. Under standard clinical scanning conditions, *i.e.* low number of counts, the COV is higher for the BPL (β=450) than the OSEM+PSF algorithm. Increase of the β-value to 550 or 700 results in a COV comparable to OSEM+PSF, however, at the cost of contrast, though still better than OSEM+PSF. At the edges of the axial field of view (FOV) where BPs overlap, COV can increase to levels at which bands become visible in clinical images, related to the lower local axial sensitivity of the PET/CT, which is due to the limited bed overlap of 23% such as advised by the manufacturer.

**Conclusions:**

The BPL algorithm performs better than the standard OSEM+PSF algorithm on small lesion detectability, SUV recovery, and noise suppression. Increase of the percentage of bed overlap, time per BP, administered activity, or the β-value, all have a direct positive impact on image quality, though the latter with some loss of small lesion detectability. Thus, BPL algorithms are very interesting for improving image quality, especially in small lesion detectability.

## Background

PET/CT scans are commonly performed to stage disease or to determine response to treatment, most often with ^18^F-FDG. However, one of the limitations of PET is its relatively poor, approximate 5 mm, spatial resolution and hence a large voxel size used, which leads to failure to detect small lesions due to an underestimation of tracer uptake [1]. In oncology imaging the use of standardized uptake values (SUVs) has a specific role in staging of disease and assessing patient response to cancer therapy. SUV is defined as the ratio between activity concentration measured within a region of interest (ROI) and the decay-corrected amount of injected activity divided by the patient weight [2]. Therefore, the use of SUVs would ideally remove variability introduced by differences in patient weight and the amount of injected ^18^F-FDG. An example of the use of SUVs in staging disease and taking subsequent treatment decisions is the Deauville score for Hodgkin lymphomas and non-Hodgkin lymphomas [3, 4] or differentiating benign from malignant lung disease [5]. However, the applicability of SUVs is still limited in daily practice because biological and technical variances are still too large to agree on a cut-off value for the SUV.

Biological variances that affect SUV variability are the patient’s blood glucose concentration, the patient’s metabolic rate, duration of the uptake phase, and correct measurement of the patient’s weight. Within most European countries, including The Netherlands, the administered dose of ^18^F-FDG to the patient can be up to a factor 2-4 times lower than commonly used in countries outside Europe, such as the USA [2]. The motives for limiting the administered activity are related to both cost reduction and radiation exposure reduction for patients and staff. A requirement to administer a patient-optimized activity instead of standard amount is to know the patient weight upon ordering. When patient weight is known in advance, the activity to be administered to the patient can be calculated using different methods, such as linear, quadratic or quadratic-like methods. These all aim to optimize image quality and acquisition time independent of patient weight, for example by keeping a constant signal-to-noise ratio (SNR) in the liver [6].

The main technical challenge in harmonizing SUV values are the different PET/CT scanners used and image reconstruction methodologies applied, which both affect accuracy and reproducibility of SUV measurements [2, 7]. Therefore, initiatives like the European EARL accreditation system help to set up a methodology to compare results of different institutes and PET/CT scanners by narrowing the SUV results and making them reproducible over time [3, 8]. New developments in the hardware of PET/CT scanners and reconstruction methods available force these EARL criteria to be updated frequently [9]. Most modern PET/CT scanners have implemented reconstruction algorithms that were developed over the last decades, applying time of flight (TOF) information and point spread function (PSF) modelling [10]. The algorithm most often applied to optimize contrast-to-noise is the ordered-subset expectation maximization (OSEM) reconstruction. However, to prevent image noise from increasing excessively iterative reconstructions like OSEM have to be stopped after 2-3 cycles and thus before full contrast convergence [10, 11]. In 2014, GE Healthcare introduced a Bayesian penalized likelihood (BPL) iterative PET reconstruction algorithm in their commercial software, coined ‘Q.Clear’, which is available on their PET/CT scanners. It includes PSF modelling and controls the noise through the use of a penalty term. Although penalized likelihood algorithms have already been developed in the 1980s [12], their clinical use in PET/CT has so far been limited. The BPL algorithm includes a relative difference penalty [13], which is a function of the difference between neighboring voxels and a function of their sum [14]. This penalty function acts as a noise suppression term and is controlled by a unit-less penalization factor (the β-value), which is the only user-input variable set in the algorithm [10, 14]. Modified block sequential regularized expectation maximization is used as an optimizer for this BPL algorithm, which, because of the penalty function, allows an effective convergence to be achieved in images, potentially providing a more accurate SUV [12, 14]. Accordingly, BPL has been shown to significantly improve signal-to-noise in clinical scans, compared with OSEM, particularly in small lesions [14–16]. A higher β-value results in stronger noise suppression, though at the loss of convergence and detectability of smaller lesions. Finding an optimal β-value in clinical practice depends on finding a balance in counting statistics and resulting image quality. Others have suggested to use β-values of 400-500 for whole-body ^18^F-FDG studies [14], 400 or 600 for pulmonary nodules evaluation [16, 17], 350-400 for brain studies [18], 300 for ^18^F-fluciclovine [19], and 4,000 for low-count ^90^Y studies [20].

The aim of this study was to determine the performance, *i.e.* contrast recovery and signal-to-noise, of BPL with different β-values compared to OSEM+PSF in ^18^F-FDG studies acquired under realistic clinical conditions using phantom- and patient-based studies.

## Methods

### PET/CT scanning and reconstruction

Scans were performed on a TOF 3-ring Discovery 710 PET/CT scanner with lutetium yttrium orthosilicate (LYSO) crystals (GE Healthcare), see [21] for an overview of the performance of the system.

Images were reconstructed using 3 different algorithms, each using low dose CT information for attenuation correction and the same normalization correction factors with scatter and randoms corrected. The standard PET reconstruction used in our center for ^18^F-FDG WB imaging is the TOF ordered-subset expectation maximization (OSEM) protocol with point spread function (PSF) (2 iterations; 24 subsets; 6.4 mm Gaussian filter; [1:4:1] axial weighted). The sinograms generated at the time of scanning were retrospectively processed to generate an OSEM reconstruction without PSF (2 iterations; 24 subsets; 6.4 mm Gaussian filter; [1:4:1] axial weighted) and with BPL reconstructions (Q.Clear, GE Healthcare; number of iterations is variable and dependent on sinogram size) [14]. BPL was studied for 3 different penalization factors (β): 450, 550 and 700. The low dose CT was obtained using a pitch of 3.27 mm, 120 kVp and auto-mAs using filtered back projection (FBP).

Multiple reconstructions of the NEMA phantom data (see below) at shorter acquisition times data were generated retrospectively (retro-reconstructions) from the list mode data acquired for 5 min/bed. The axial PET field of view (FOV) length is 15.7 cm; one bed position (BP) contains 47 slices, and a BP overlap contains 11 slices (overlap of 23%).

### Phantoms

A standard National Electrical Manufacturers Association (NEMA) NU2-2007 image quality phantom [22] and a modified Micro Hollow Sphere (MHS) phantom [23] with an extra central 10 mm sphere were scanned head-to-head in 3 bed positions on our PET/CT scanner, with the spheres of the NEMA phantom centered at peak sensitivity of a bed position. The NEMA phantom has a 5 cm diameter cylindrical lung insert in the center and six fillable spheres with internal diameters of 10, 13, 17, 22, 28 and 37 mm, positioned around the lung insert. The lung insert is filled with polystyrene beads to mimic lung tissue density. The MHS phantom has five fillable spheres with internal diameters of 4, 5, 6, 8 and 10 mm, the first four positioned equiradially around the largest 10 mm sphere. The phantom background compartment and all spherical inserts were filled with ^18^F-FDG solutions aimed at activity concentrations of 1 kBq/ml and 10 kBq/ml respectively, at the start of the measurements, resulting in an initial sphere-to-background activity concentration ratio of 10-to-1. Thus, all spheres are ‘hot’. To accomplish a 4-to-1 and 2-to-1 sphere-to-background ratio the background was filled with extra activity to perform sequential scans 50 min and 100 min after the first scan of the phantoms, which had a 10-to-1 ratio. Activity concentrations at the start of the measurement were 6.5 kBq/ml and 1.6 kBq/ml (4-to-1) and 4.8 kBq/ml and 2.6 kBq/ml (2-to-1), respectively. The phantoms were scanned for 5 minutes (10-to-1 ratio), 7 minutes and 10 seconds (4-to-1 ratio) or 10 minutes (2-to-1 ratio) using the protocols in the EANM/EARL guidelines [1, 8, 24].

Images of the NEMA phantom were analyzed using the EARL analysis tool with a VOI_50_ isocontour (3D isocontour at 50% of maximum pixel value, corrected for background uptake [1, 24]) and data of the MHS phantom were analyzed by a custom-written macro in FIJI [25] giving results similar to the EARL analysis tool. The following image quality parameters were determined: contrast recovery, background variability, residual lung error and the coefficient of variation (COV) for phantom or noise levels for patient studies. Methods for determining the first three parameters are defined in the EARL guidelines using the current min-max accreditation limits [8]. The COV were determined by dividing the standard deviation (SD) by the mean pixel value within the ROI using FIJI for all PET slices of a BP. The background ROI was drawn in the background compartment of the phantom as defined in the Results section (Fig. 4b). The residual lung error was determined using the lung insert of the NEMA phantom.

### Clinical evaluation

^18^F-FDG PET/CT patient scans were acquired on the same PET/CT scanner used for the evaluation of the phantoms. Patients were required to fast for at least 4 hours before their scan. Their blood glucose was measured before intravenous injection of ^18^F-FDG and was found to be below 12 mmol/liter. Administered patient dose was according to equation 1; a scanner optimized form of the formula published in [24] according to the steps described in [6]:

1 $$ {\mathsf{A}}_{\mathsf{FDG}}\left[\mathsf{MBq}\right]=\mathsf{0.027}\left[\mathsf{MBq}/\mathsf{k}{\mathsf{g}}^{\mathsf{2}}\right]\times {\mathsf{W}}^{\mathsf{2}} $$

with W weight in kg, which results in a constant SNR for the liver [6]. A minimum dose of 120 MBq was administered to the patients. Patients were imaged 60 ± 3 min after injection. Scans were performed from skull base to knees or feet (whole body), using standard clinical protocol. The PET images were acquired under normal tidal respiration for 2½ min per bed position for the torso and for 1 min per bed position for the legs, because of lower attenuation locally. Using FIJI, mean background activity and SD were measured per slice drawing a ROI in muscle tissue with exclusion of large blood vessels containing an higher activity concentration

For our study, eight ^18^F-FDG PET/CT scans were retrospectively selected of patients that received a dose of less than 160 MBq, who were scanned for at least 2 bed positions at the legs with 1 min/bed. These studies were acquired between June and September 2017 for evaluation of oncology (6 studies) and inflammation (2 studies). Studies were selected of 7 women and 1 man with a weight range of 44-75 kg and a median weight of 61 kg.

Informed consent is not required in our institution for retrospective reviews of the kind of studies described in this paper; data were anonymized before analysis.

## Results

### Improved contrast recovery with BPL in phantom studies

**Fig. 1:**
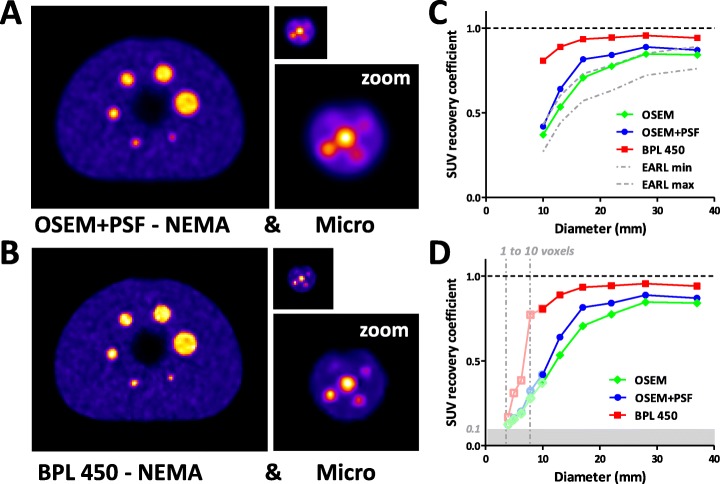
Results of phantom studies with a sphere-to-background ratio of 10-to-1. ^18^F-FDG PET/CT imaging of the standard NEMA image quality phantom and micro hollow sphere phantom (real size and zoom) having different sized spheres ranging in size from 4-37 mm (0.03-27 ml). Images were reconstructed with **a**.) OSEM+PSF, or **b**.) BPL algorithm with β=450. **c**.) The SUV_mean_ recovery coefficients of OSEM, OSEM+PSF and BPL 450 reconstructions of the NEMA phantom, compared to the EARL standard. **d**.) SUV_mean_ recovery coefficients of OSEM, OSEM+PSF and BPL 450 reconstructions extended by analysis of the micro phantom. Micro phantom data were extrapolated based on 10 mm sphere results present in both phantoms as a way to correct for attenuation (uncorrected data in Table 1 and Figure 2). “0.1” represents the level at which SUV recovery is the same as background (gray area). For the smallest spheres the volume equivalent in voxels is indicated (dash-dotted lines)

To get a standardized overview of differences between a regular OSEM reconstruction and a BPL reconstruction with β = 450, phantom experiments were performed over a broad range of spherical lesion sizes from 4-40 mm. Spheres were filled with ^18^F-FDG at a 10-to-1 ratio of sphere-to-background, mimicking high intensity ^18^F-FDG uptake in clinical studies [26]. The axial slice taken at the maximum diameter of the spheres using OSEM+PSF (Fig. 1a) or BPL 450 (Fig. 1b) clearly shows a better recovery of small spheres (8-13 mm) when BPL 450 is used. Quantification of the results of the NEMA phantom by measuring the mean standard uptake value (SUV_mean_) shows that using OSEM without PSF leads to a performance of the system well within the current EARL min-max limits (Fig. 1c) [8]. Using PSF leads to an overall improved SUV recovery, especially for medium-sized spheres of 13-22 mm (see also Table 1). Application of BPL 450 leads to a higher SUV recovery for all spheres. Particularly smaller spheres of 10-17 mm show a higher recovery rate. Analysis of the micro hollow sphere phantom data shows that the improved SUV recovery using BPL is maintained down to a sphere size of 5 mm (Fig. 1d), whereas using an OSEM reconstruction performance is up to a factor 3 less and independent of using PSF.

**Table 1:** SUV recovery values for different sphere-to-background ratios^a^

Sphere size (mm)	10-to-1 ratio					4-to-1 ratio					2-to-1 ratio				
	OSEM	OSEM + PSF	BPL 450	BPL 550	BPL 700	OSEM	OSEM + PSF	BPL 450	BPL 550	BPL 700	OSEM	OSEM + PSF	BPL 450	BPL 550	BPL 700
NEMA^b^															
*37*	0.842	0.870	0.941	0.934	0.926	0.914	0.943	0.979	0.971	0.961	0.971	0.986	1.009	0.998	0.989
*28*	0.847	0.888	0.956	0.951	0.945	0.862	0.902	0.959	0.955	0.947	0.985	1.013	1.042	1.031	1.015
*22*	0.775	0.841	0.944	0.934	0.925	0.856	0.930	1.011	0.998	0.975	0.931	0.968	1.009	0.985	0.952
*17*	0.707	0.815	0.934	0.927	0.913	0.772	0.852	1.010	0.977	0.934	0.845	0.866	0.910	0.871	0.819
*13*	0.534	0.640	0.889	0.854	0.807	0.569	0.558	0.708	0.660	0.601	0.650	0.642	0.646	0.631	0.617
*10*	0.370	0.419	0.807	0.718	0.624	0.386	0.390	0.402	0.387	0.372	0.647	0.648	0.647	0.627	0.612
Micro^b^															
*10*	0.432	0.523	0.876	0.836	0.817	0.470	0.536	0.769	0.741	0.703	0.681	0.706	0.728	0.717	0.699
*8*	0.320	0.396	0.837	0.808	0.764	0.342	0.380	0.560	0.522	0.478	**0.588**	**0.572**	**0.555**	**0.564**	**0.560**
*6*	0.210	0.234	0.414	0.389	0.356	0.293	0.298	0.329	0.319	0.310	**0.589**	**0.574**	**0.551**	**0.554**	**0.555**
*5*	0.167	0.182	0.331	0.304	0.268	**0.266**	**0.260**	**0.272**	**0.268**	**0.265**	**0.563**	**0.550**	**0.512**	**0.519**	**0.529**
*4*	**0.132**	**0.133**	0.174	0.157	0.153	**0.272**	**0.266**	**0.266**	**0.268**	**0.265**	**0.544**	**0.534**	**0.487**	**0.492**	**0.512**

^a^If SUV recovery values are (almost) equal to background and thus invisible (in boldface)

^b^Indicated are the type of phantom used, i.e., the NEMA or micro hollow sphere phantom

**Fig. 2:**
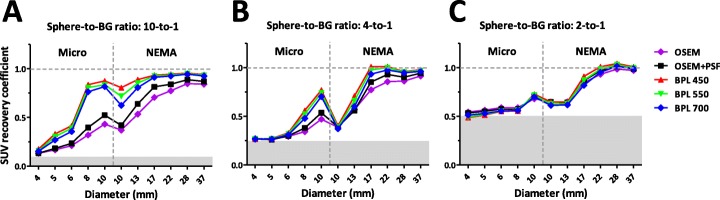
Results of phantom study with different sphere-to-background ratios. SUV_mean_ recovery coefficients of all micro and NEMA spheres reconstructed with different algorithms; OSEM, OSEM+PSF and BPL with β = 450, 550 and 700. Results are shown for all three different sphere-to-background ratios: **a**.) 10-to-1, **b**.) 4-to-1, and **c**.) 2-to-1. Gray areas indicate levels at which SUV recovery is the same as background

Similar experiments were performed with phantoms filled with a sphere-to-background ratio of 4-to-1 and 2-to-1; representing moderate and low intensity uptake in clinical studies, respectively. These 4-to-1 and 2-to-1 sphere-to-background ratios show that BPL 450 results in a better SUV recovery than OSEM, although this effect is less distinct than for a 10-to-1 ratio (Table 1 and Fig. 2). Moreover, BPL reconstructions with a higher β of 550 and 700 were analyzed, which show a somewhat lower SUV recovery at increasing β-value (Fig. 2). Next to SUV recovery, another clear difference between OSEM and BPL reconstructions is a lower residual lung error for the latter (Table 2), which is hardly influenced by the sphere-to-background ratio and only slightly by background activity.

**Table 2:** Residual lung error

Reconstruction type	10-to-1 ratio 1.0 kBq/ml BG activity	4-to-1 ratio 1.6 kBq/ml BG activity	2-to-1 ratio 2.6 kBq/ml BG activity
*OSEM w/o PSF*	9.0 ± 1.5 %	8.3 ± 0.6 %	7.6 ± 0.4 %
*OSEM with PSF*	8.7 ± 1.1 %	8.2 ± 0.7 %	7.7 ± 0.4 %
*BPL 450*	2.4 ± 0.4 %	2.2 ± 0.4 %	1.7 ± 0.3 %
*BPL 550*	2.6 ± 0.5 %	2.4 ± 0.4 %	2.0 ± 0.3 %
*BPL 700*	2.9 ± 0.5 %	2.7 ± 0.4 %	2.3 ± 0.4 %

### Improved contrast recovery with BPL in patient studies

**Fig. 3:**
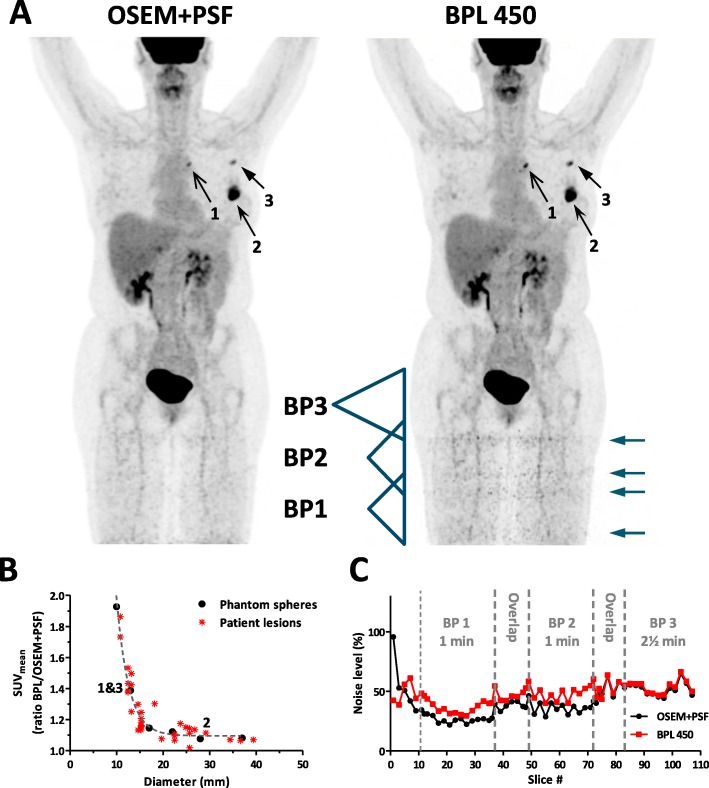
Lesion detectability and noise levels per slice in patient studies. **a**.) Representative ^18^F-FDG PET/CT patient study reconstructed by OSEM+PSF and BPL 450. Indicated are three lesions of different sizes (numbered arrows) and the position of three BPs, sensitivity (indicated by the triangle), and their overlap region. The acquisition times are different: BP1 & BP2 (1 min/bed) and BP3 (2½ min/bed). ‘Noise bands’ in the legs are indicated by arrows. **b**.) Ratio of the SUV_mean_ of *N* = 33 lesions from *N* = 8 patients measured in reconstructions from BPL 450 and OSEM+PSF. In addition, the ratios are given for the 6 spheres of the NEMA as measured in Fig. 1c. The three lesions of *A.)* are indicated in the graph. A single exponential decay was fitted to both data sets showing an agreement of *R*^*2*^ = 0.917. ***c****.)* Noise levels per slice, defined as the SD/mean of a ROI drawn in muscle tissue, are given for the 3 BPs of the patient scan indicated in *A.)*. Acquisition times per BP as well as overlap regions of 11 slices between two BPs are indicated

Analysis of the SUV_mean_ of different sized lesions in ^18^F-FDG patient studies (*N* = 8) reconstructed with OSEM+PSF or BPL 450 revealed that for the latter reconstruction SUV recoveries are higher, in particular for smaller lesions (Fig. 3a-b). The observed ratio between SUV_mean_ of BPL and OSEM+PSF for different sized lesions in patients is similar (*R*^*2*^ = 0.92) to those observed for the spheres of the NEMA phantom (Fig. 1c). Thus, both in phantom as well as in patient studies smaller lesions are recovered better using BPL.

### Noise ‘bands’ at overlapping bed positions

Looking into ^18^F-FDG patient studies our physicians noticed an artefact at the legs of some studies, which is more pronounced when a BPL 450 algorithm is used than with the OSEM+PSF algorithm. This artefact manifests as noisy ‘bands’ for bed positions taken in the legs (Fig. 3a). Optimization of the total study time at our institute, based on earlier OSEM+PSF studies lead to an acquisition time of 1 min/bed for the legs, whereas bed positions for the rest of the body are acquired at 2½ min/bed. To analyze the noise, we measured the noise levels in muscle tissue per slice within 3 bed positions of one study (Fig. 3c). Clearly when the acquisition time is only 1 min (BP1 & BP2) the noise levels in slices reconstructed with BPL is higher than when OSEM+PSF is used, whereas this effect is vanishing when acquisition time is 2½ min per bed. Secondly, the noise levels are stronger at the edges of overlapping bed positions, where the sensitivity is smaller, which results in double ‘bands’ in patient studies (Fig. 3a).

**Fig. 4:**
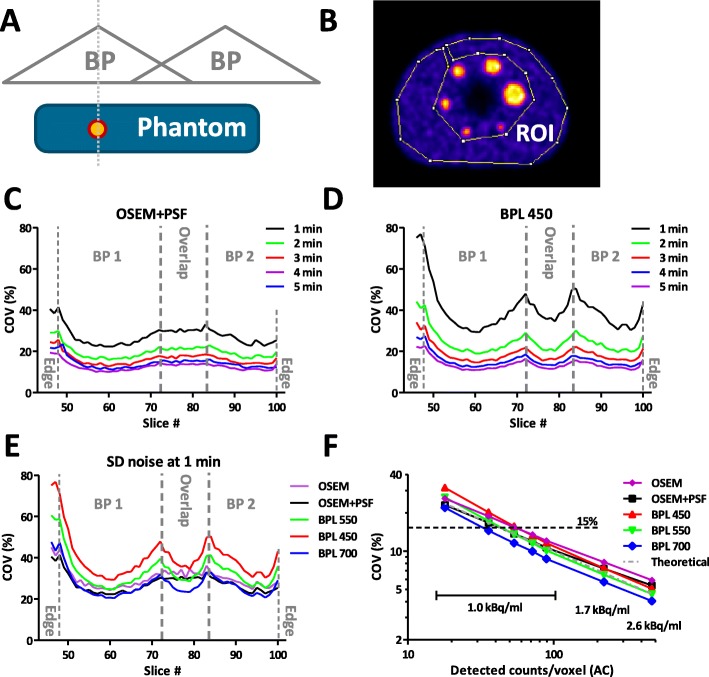
COV variation within the BPs. **a**.) Positioning of the NEMA phantom and the spheres within 2 BPs. Spheres are oriented such that they are the maximum of the sensitivity of a BP. **b**.) Region of interest (ROI) drawn in the NEMA phantom excluding the spheres used for background measurements of mean and SD values to calculate COV. **c**-**d**.) COV for different slices within 2 BPs and its overlap region with different acquisition times varying between 1 and 5 min/bed of the phantom filled at a 10-to-1 ratio (BG activity: 1.0 kBq/ml). Shown are the results of ***c****.)* OSEM+PSF reconstructions and **d**.) BPL 450 reconstructions. **e**.) Comparison of the COV for different slices at 1 min/bed and using an OSEM+PSF or BPL 450, 550 and 700 reconstructions. **f**.) Relation between the observed noise and the amount of counts detected per voxel based on PET sensitivity of the 11 central slices of a BP and known activity of the background. The first 5 points on the x-axis correspond to 1 to 5 min/bed. Two extra points were extracted from background measurements based on the 4-to-1 and 2-to-1 phantom measurements; BG activity: 1.7 kBq/ml and 2.6 kBq/ml, respectively

To study this effect in more detail, the coefficients of variation (COV) in slices of the phantom studies were analyzed for different simulated acquisition times varying between 1 and 5 min (Fig. 4). The mean background activity and SD of all slices were measured in the large NEMA phantom in which an overlap region between two bed positions is present (Fig. 4a-b). Longer acquisition times result in less noise for all reconstruction methods studied (Fig. 4c-d). Secondly, the COV increases when moving away from the center of a bed position to the overlap region. Here, the axial sensitivity is locally lower. Within the overlap area, however, we expect to see only random variations and no structural ones, because the combined sensitivity of the two bed positions is constant within this overlap area. We indeed observe this for the OSEM+PSF reconstruction. Using BPL, however, a drop in the COV can be observed at the center of the overlap.^1^

### Varying the BPL β-value to reduce noise

**Fig. 5:**
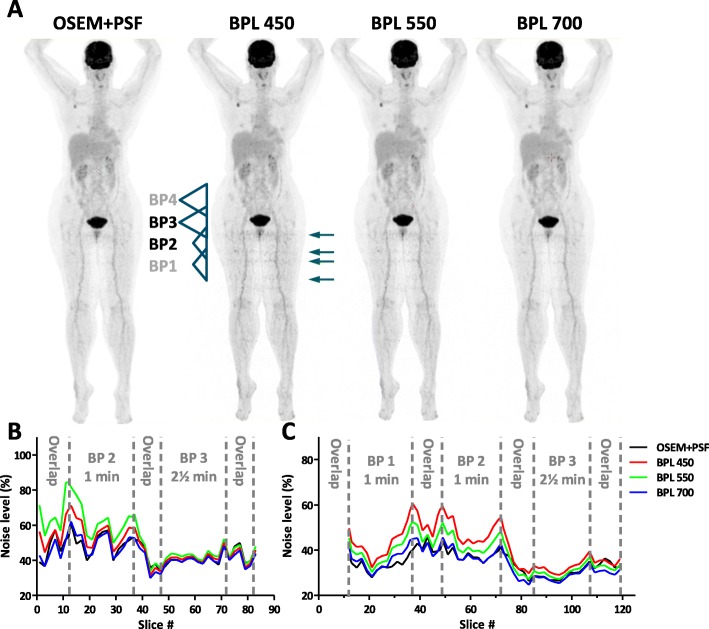
Comparison of noise levels in patient studies. **a**.) Representative ^18^F-FDG PET/CT patient study reconstructed by OSEM+PSF and BPL with a β of 450, 550 and 700. The two BPs are indicated at which acquisition time changes from 1 min/bed (BP2) to 2½ min/bed (BP3). **b**.) Noise levels per slice at the BPs of the study shown in *A.)*. Indicated are the BPs and overlap regions. **c**.) Averaged noise levels of multiple patient studies (*N* = 8) at the transition of BPs taken at 1 min/bed (BP1 & BP2) to 2½ min/bed (BP3)

By increasing the noise penalty factor β of the BPL algorithm we should be able to obtain reconstructed images that are less noisy. Indeed, reconstructions of the NEMA phantom at 1 min with a β-value of 550 or 700 are less noisy than those with a β-value of 450 (Fig. 4e), with those with a β of 700 having a COV comparable to OSEM+PSF. Retrospectively reconstructed ^18^F-FDG patient studies show also that with a β of 550 and 700 the noise levels in the legs (1 min/bed acquisition time) are reduced (Fig. 5a). Slice-by-slice analysis of the bed positions at the transition of 1 min/bed (BP2) to 2½ min/bed (BP3) reveals that by rising β noise levels are reduced, with similar noise levels of OSEM+PSF and BPL using a β of 700 (Fig. 5b). This effect is most pronounced at the BPs of 1 min/bed.

Eight patient studies in total were analyzed and the noise levels per slice of every patient study were averaged within three bed positions to average out inhomogeneities due to patient anatomy. Figure 5c shows the results of two bed positions with an acquisition time of 1 min/bed (BP1 & BP2) and one of 2½ min/bed (BP3) including overlap regions. These data show clearly that moving away from the center of a bed position and at the edges of a bed position overlap, the noise levels are strongest. These patient study findings agree well with the findings in our phantom studies (Fig. 4e). The noise level behavior for the different algorithms can be explained on the basis of Poisson statistics, for which the characteristic relation between detected counts per voxel and COV is given in Figure 4f, which will be further discussed below.

## Discussion

This study addressed the relationship between contrast recovery and the COV under conditions of low counts while applying a BPL algorithm. The investigated BPL algorithm Q.Clear by GE Healthcare is available on their newer Discovery PET/CT scanners. BPL algorithms have been investigated before by other groups, however, not combining measurements of a NEMA and small sphere phantom while varying imaging acquisition times per BP. In addition, studying the correlation between COV and detector sensitivity within overlapping bed positions is new. In clinical PET/CT imaging, the choices to be made when optimizing scanning protocols depend on the primary goal: whether it is to achieve shorter imaging times, decreased radiation dose, or improved image quality and lesion detectability. In practice, it will always be a trade-off between these factors. Our study confirmed that in order to have similar or better noise reduction, the β-value of the BPL algorithm needs to be higher than 450 to have an optimal result, in particular under conditions of low counts. In addition, our results suggest to use a BPL algorithm with an optimized β-value, instead of a traditional OSEM+PSF algorithm.

To support this, we compared the application of a BPL algorithm to OSEM with and without PSF in phantom as well as in patient studies. As reference to start our optimization the scanning and post-processing settings recommended by the manufacturer were used. Our study confirmed that PSF for OSEM indeed has a positive effect on SUV recovery and COV. For BPL, where the only user adaptable setting is the β-value, we showed that increasing the β from 450 to 550 and 700 reduces noise. However, this comes with somewhat less small lesion detectability and lower SUV recovery values (Figs 1-2). Other studies showed that lowering β-values down to 100 has a positive effect on contrast recovery though with higher noise levels, in general they conclude that for ^18^F-FDG whole body scans a β-value of 400-500 would be optimal [14, 16, 27]. We showed that SUV recoveries achieved using BPL with β-values of 450 to 700 always exceed those achieved by OSEM with or without PSF. In support of our findings others already showed that SUV recoveries achieved when optimizing OSEM+PSF do never reach the same levels as achieved using BPL [27]. With phantom studies with spheres sized 4-37 mm, we demonstrated improved small lesion detectability for spherical lesions down to 5 mm in diameter due to high levels of convergence achieved with BPL, which extends the findings of an earlier phantom study with spheres sized 10-37 mm [14]. In addition, we show that this effect only holds for medium (4-to-1) and high (10-to-1) sphere-to-background ratios. Moreover, this contrast enhancement effect was also seen for patient lesions, which complies to earlier studies [14–16]. The improved detectability by BPL of lesions sized 8-13 mm seems of special clinical interest, because these lesions would be sometimes missed using OSEM+PSF. In addition, spill-in or spill-out of counts is also effectively reduced using BPL, as demonstrated by lower SUV levels seen in the lung insert in the phantom, which is in line with an earlier study by Teoh et al. [14]. Therefore, we expect in the coming years that, besides hardware developments like digital TOF, post-processing developments like the implementation of BPL algorithms will enhance the performance of PET at a similar pace as we have seen the last decade. Likely, the EANM will have to continue updating the EARL min-max accreditation limits to keep up with these developments, as they did over the last decade [8, 9].

Within Europe, as well as in many countries outside Europe, radiation dose to the patient is an important optimization factor. Therefore the activity administered to the patient is limited, which next to being beneficial for dose reduction, is also economically beneficial due to lower costs for radiopharmaceuticals. On the other hand, the total imaging time per patient study should be limited for patient comfort and optimal scanner throughput. Optimizing acquisition time per bed position for different anatomical regions is common practice for whole body PET/CT studies. For example, when scanning the legs, multiple bed positions are needed. However, attenuation is much less than in the torso, and PET images of the legs have, in general, less clinical value. Therefore, acquisition times per bed position for the legs section are, at many institutes, set a factor 1.5-3× lower than for the rest of the body, which brings down the total scan time per patient with 5-10 minutes. On the other hand, images should still be of a quality high enough to see anomalies with enough detail, and noise levels should be acceptable and show no artefacts.

SNRs in PET images depend on the number of desintegrations detected by the crystals in individual voxels of the PET/CT detector. These detectors have a 3D sensitivity profile that linearly drops from the center, thus COV at the edges of the axial field-of-view (FOV) will be higher than in the center. To improve counting statistics, and thus image quality, BPs need to overlap. The Poisson noise at a voxel level relates to the detected counts (N) as 1/√N. For BP overlap regions noise would correlate with accumulated counts, and thus, the COV per slice correlates to the sensitivity profile of overlapping BPs. The Discovery 710 scanner has an default overlap of 23%, as recommended by the manufacturer. In our studies at 1 min/bed, we observed the appearance of bands in the PET images. These bands have a 1.3× higher COV at the edges of the overlap region than in the center of the overlap region (average local sensitivity 83%), and correlates to regions where the local sensitivity is 50%. The default choice of a 23% overlap, as recommended by the manufacturer, was made due to the axial FOV of the system of only 15.7 cm; several other systems have a longer axial FOV [28]. For patient comfort, it was decided to have an overlap smaller than 50% and thus less BPs, instead of more overlap and more BPs. Most often, other vendors advise to use a higher overlap up to 50%. Under these conditions, overall scan time per patient can be maintained by reducing acquisition time per BP [24]. Another benefit will be reduced differences between center and edge positions and thus result in smoother images. Therefore, we suggest to increase the bed overlap or increase the acquisition time per bed in our center.

An alternative to bring down the COV is to increase the β-value; we show that a β of 700 can effectively reduce the COV in the image to acceptable levels. Although at the same time it slightly compromises lesion detectability and SUV recovery. The EARL guidelines state that an optimal COV for exact quantification of the SUV in phantom studies should be below 15% [29]. From Figure 4f we may conclude that to comply with a COV < 15% the scan time per bed (counting statistics) or the administered activity should be increased. When making a whole body scan with variable scan times per bed, β-value modulation to balance SUV recovery and COV would be an option worth considering in future reconstruction software for PET/CT scanners.

## Conclusion

Performance of the BPL algorithm is superior to the standard OSEM+PSF algorithm in small lesion detectability, SUV recovery and COV suppression. However, when applying BPL on PET/CT images acquired with low counting statistics, noise levels at the edges of a bed position can increase strongly and result in visible bands. Slightly increasing the β-value, the percentage of bed overlap, time per bed position or administered activity might reduce the noise, however, at the cost of increased scan time, radiopharmaceutical costs or small lesion detectability. Therefore, for the type of ^18^F-FDG studies described in this study we would suggest to increase the β-value to 500-600 and consider increasing the bed overlap for those scanners that use a very low default value of 23% overlap.

## Data Availability

The datasets analyzed during the current study are available from the corresponding author on reasonable request.

## Abbreviations

^18^F-FDG: Fludeoxyglucose (^18^F)
BP: Bed position
BPL: Bayesian penalized likelihood
COV: Coefficient of variation
EANM: European Association of Nuclear Medicine
EARL: EANM Research Ltd
FOV: Field-of-view
LYSO: Lutetium yttrium orthosilicate
MHS: Micro hollow sphere
NEMA: National Electrical Manufacturers Association
OSEM: Ordered-subset expectation maximization
PET/CT: Positron emission tomography / computed tomography
PSF: Point spread function
ROI: Region of interest
SNR: Signal-to-noise
SUV: Standard uptake value
TOF: Time-of-flight
VOI: Volume of interest

## Footnotes

1 An explanation for this observation was sought by contacting and discussing it with the manufacturer and developer of the BPL algorithm. However, together we found no plausible explanation. Maybe future studies can address this issue
